# The flat-plate plant-microbial fuel cell: the effect of a new design on internal resistances

**DOI:** 10.1186/1754-6834-5-70

**Published:** 2012-09-21

**Authors:** Marjolein Helder, David PBTB Strik, Hubertus VM Hamelers, Cees JN Buisman

**Affiliations:** 1 Wageningen University - Sub-department of environmental technology, PO box 17, Wageningen 6700 AA, The Netherlands; 2 Wetsus– Centre of excellence for sustainable water technology, Agora 1, Leeuwarden, The Netherlands

**Keywords:** Plant-microbial fuel cell, Design, Flat-plate, Internal resistance, Root growth, *Spartina anglica*, Sustainable electricity

## Abstract

Due to a growing world population and increasing welfare, energy demand worldwide is increasing. To meet the increasing energy demand in a sustainable way, new technologies are needed. The Plant-Microbial Fuel Cell (P-MFC) is a technology that could produce sustainable bio-electricity and help meeting the increasing energy demand. Power output of the P-MFC, however, needs to be increased to make it attractive as a renewable and sustainable energy source. To increase power output of the P-MFC internal resistances need to be reduced. With a flat-plate P-MFC design we tried to minimize internal resistances compared to the previously used tubular P-MFC design. With the flat-plate design current and power density per geometric planting area were increased (from 0.15 A/m^2^ to 1.6 A/m^2^ and from 0.22 W/m^2^ to and 0.44 W/m^2^)as were current and power output per volume (from 7.5 A/m^3^ to 122 A/m^3^ and from 1.3 W/m^3^ to 5.8 W/m^3^). Internal resistances times volume were decreased, even though internal resistances times membrane surface area were not. Since the membrane in the flat-plate design is placed vertically, membrane surface area per geometric planting area is increased, which allows for lower internal resistances times volume while not decreasing internal resistances times membrane surface area. Anode was split into three different sections on different depths of the system, allowing to calculate internal resistances on different depths. Most electricity was produced where internal resistances were lowest and where most roots were present; in the top section of the system. By measuring electricity production on different depths in the system, electricity production could be linked to root growth. This link offers opportunities for material-reduction in new designs. Concurrent reduction in material use and increase in power output brings the P-MFC a step closer to usable energy density and economic feasibility.

## Background

With a growing world population and increasing welfare, energy demand worldwide is increasing [1]. Currently used fossil fuels are unevenly distributed over the world, are being depleted, and are unsustainable [2,3]. Sustainable alternative energy sources that are available nowadays all have their drawbacks. They are weather dependent (wind, solar power), compete with food/feed production (some biofuels) [3,4] or involve high investment costs [5]. The Plant-Microbial Fuel Cell (P-MFC) is a technology that can potentially be used weather-independent, at any place in the world where plants can grow, without competition with food or feed production, and with relatively low investment costs [6-11]. The technology is still in its infancy, however, and large improvements should be achieved to make this technology energetically and economically feasible. One of the main challenges with current state of technology is its power output. Even though theoretical power output is estimated at 3.2 W/m^2^ geometric planting area [10],maximum power output has been improved only from 65 mW/m^2^ in 2008 [12] to 220 mW/m^2^ in 2010 [9] in systems with plants as sole organic matter source. Current biomass-electricity systems, like anaerobic digestion, produce the same amount of electricity per geometric planting area as the maximum that was achieved in P-MFCs, 220 mW/m^2^[10]. So even at current power output, the P-MFC could compete with anaerobic digestion based on electricity production per geometric planting area. But maximum power outputs of the P-MFCs have been achieved in short term tests like polarization curves and were not sustained for longer periods of time. Over longer periods of time, average power output is limited to maximally 50 mW/m^2^ geometric planting area [11]. In some experiments a decrease in power output during runtime of an experiment was observed,due to an increase of membrane resistance and build-up of ion-transport resistances [11]. In one recent publication, however, it is shown that average power output increases with runtime of the experiment. This latter experiment was done with a flat-plate P-MFC [13].

Power density of a P-MFC is determined by several aspects of the system: solar radiation, photosynthetic efficiency of the plant, organic matter allocation from plant to soil, and efficiency of the Microbial Fuel Cell (MFC) [10]. Timmers et al. identified that the P-MFC has a high internal resistance, which limits the power density [14]. In order to increase the power density, internal resistance should be decreased. When calculating the power density as a function of the internal resistance,

(1) $$P=V_{\max}\times i-\left(i^{2}\times R_{i}\right)$$

Equation 1 Power density (P) as function of maximum voltage (V_max_ = 1.1 V), current density (i,A/m^2^) and internal resistance (R_i_, Ωm^2^) in a P-MFC [15,16]. Derivation of equation can be found in supporting information.

**Figure 1:**
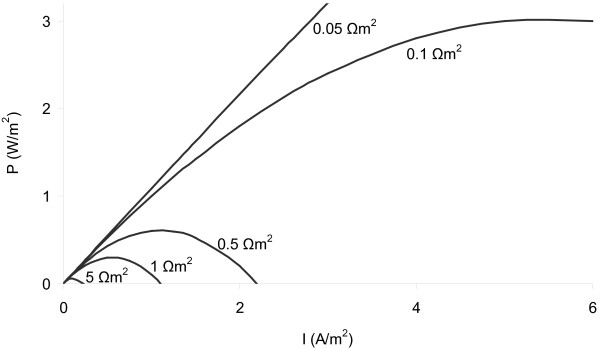
**Power density as a function of current density at different internal resistances, which shows that internal resistance should be <0.094 Ωm**^**2**^** to achieve a power density of 3.2 W/m**^**2**^.

It shows that current and power densities are very dependent on internal resistance of the system. From Equation 1it can be calculated that the internal resistance needs to be reduced to 0.094 Ω m^2^ membrane surface area to be able to reach the theoretical power output of 3.2 W/m^2^ membrane surface area (Figure 1).

**Figure 2:**
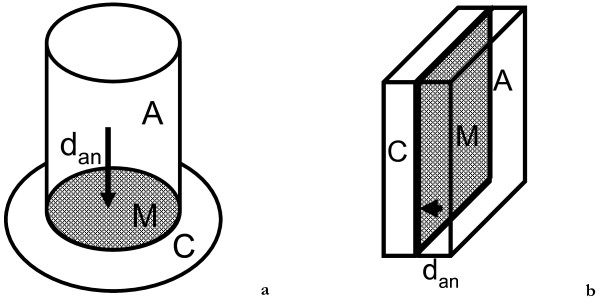
**Tubular (2a) and flat-plate (2b) design of a Plant Microbial Fuel Cell, in which A = anode, C = cathode, M = membrane, d**_**an**_ **= average distance between anode and membrane.** Distance between anode and membrane is shorter in flat-plate design than in tubular design.

One of the factors influencing internal resistance is the average distance between anode and cathode. The design that was used by Strik et al. [12], Timmers et al. [11] and Helder et al. [9] is a tubular design. In this design anode is tubularly shaped with a membrane at the bottom of the tube. Cathode is situated underneath the anode (Figure 2a). When electrons are homogeneously produced in the anode-compartment, average transport distance for a proton to travel from anode through the membrane to the cathode is relatively long. A long distance from anode to cathode leads to transport losses in the anode. Research by Timmers et al. [11] has shown that internal resistance of the P-MFC, especially transport resistance, is an important limiting factor in the power output of the system. In the flat-plate P-MFC (Figure 2b) anode and cathode are placed next to each other with a membrane vertically in between. This way distance from anode through cathode is smaller and transport resistance will be limited.

The flat-plate system has been described before, but internal resistances of this design were not characterized. In this paper we describe the different partial internal resistances in the flat-plate P-MFC after 320 days runtime of the experiment. Internal resistances were determined at different rooting depths in the P-MFC. To enable this, three separate anodes were used in the system, which were not electronically connected.

## Results and discussion

**Figure 3:**
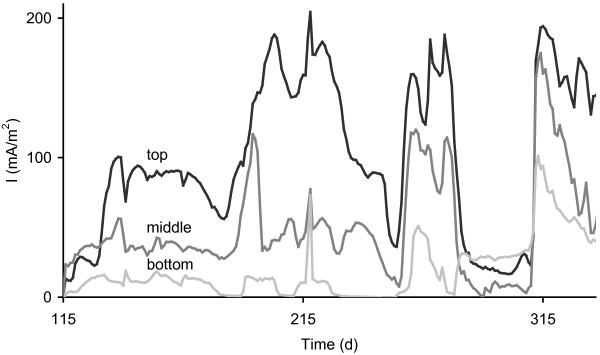
**Electricity production (mA/m**^**2**^**) in top, middle and bottom sections of P-MFC 1 during day 115 through 337 in which top section produces most electricity.** Fluctuations in power output over time are caused by changes in medium composition as described in Helder et al. 2011 [13].

The plants in both P-MFCs kept growing throughout the experiment, allowing us to acquire data during 350 days on the performance of the P-MFCs with growing plants. During the experiment top anode of the P-MFC produced most electricity, the middle part less and the bottom part the least (Figure 3). This was consistent with the root-growth. Most roots were found in the top anode (43.8 and 15.1 g in P-MFC 1 and 2), less in the middle anode (1.0 and 3.2 g) and least in the bottom anode (<0.1 and 0.1 g). Electricity production seems linked to root growth, the more roots present in the anode, the more electricity is produced. This is consistent with the idea that the organic matter used for electricity production originates from the roots.

### Anodic resistance is the highest partial internal resistance in flat-plate P-MFC

**Figure 4:**
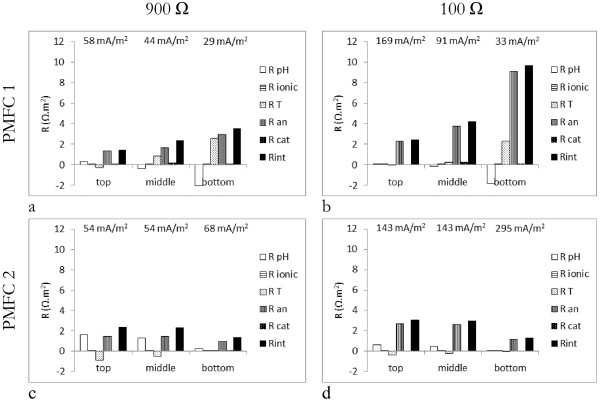
**Partial and total internal resistances (Ω.m**^**2 **^**membrane surface area) at three different depths (top, middle, bottom) in two P-MFCs on day 320 of experiment at 900 Ω and 100 Ω external resistance.**

Partial and total internal resistances were calculated for the three different levels in two P-MFCs (top, middle, bottom) on day 320 of the experiment (Figure 4). In both P-MFCs at all levels and both at low and high current density anodic resistance added substantially to the total internal resistance (Figure 4). It should be noted that we used ferric-cyanide as final electron acceptor in the cathode, thus cathode over-potential would not show in our calculations. Overpotential at the anode is the amount of energy that is lost in the oxidation of organic matter and includes activation energy, microbial energy for maintenance and growth,ohmic losses and concentration losses [16,17]. Concentration losses are determined by the substrate availability at the anode-surface and accumulation of products at the anode-surface [16,17]. Substrate availability is determined by the substrate-input and its mass transfer. Substrate-input in the P-MFC is determined by root exudation and dead root turnover [10]. It can therefore be expected that highest substrate-availability is where most roots are. Highest anodic resistance would therefore be expected where least roots are present.

In case of P-MFC 1 this was true; most roots were found in the top part of the system, less in the middle part and least in the bottom part. Anodic resistance of the bottom part of P-MFC 1 (Figure 4a, b) increases with a lower external resistance, while current density does not increase for this level. This is likely due to the fact that hardly any roots were present (<0.1 g) and so substrate was not available to increase current density. If current density cannot be increased, but external resistance allows more current to flow, overpotential will increase. The same occurred in the middle part of P-MFC 1, albeit to a lesser extent, and even in the top part of P-MFC 1, where most roots were found, anode-resistance increased with a lower external resistance, indicating possible substrate-limitation.

P-MFC 2 shows a different pattern (Figure 4c, d). Top and middle part of P-MFC 2 have shown for the largest part of the runtime of the experiment the same voltage and anode and cathode potentials. Likely an electrical connection between top and middle anode of P-MFC 2 existed, so that these two anodes have actually functioned as one. Top and middle level of P-MFC 2 show therefore the same partial and total internal resistance, even though more roots were present in the top part of P-MFC 2 than in the middle part of P-MFC 2. Since electrons could flow freely between the two anodes, substrate limitation at one of them will not directly lead to a higher anode resistance as long as at the other anode substrate is still available. Opposite to the situation in P-MFC 1, the bottom part of P-MFC 2 shows highest current and lowest internal resistance of the three levels in P-MFC 2. Like in P-MFC 1, the bottom part of P-MFC 2 hardly contained any roots. The bottom part of P-MFC 2, however, did contain at least one root-tip. Literature describes that so-called hotspots can occur in the rhizosphere [18,19]. A hotspot is a place in the root zone where microbial activity and exudation are enhanced compared to the rest of the rhizosphere [18]. Intensity of turnover processes in these hotspots is at least one order of magnitude higher than in the bulk-soil [18]. Even so, hotspots will likely have occurred in the top and middle anode as well. More research should be done to further explore the hotspot hypothesis and exclude artifacts.

**Figure 5:**
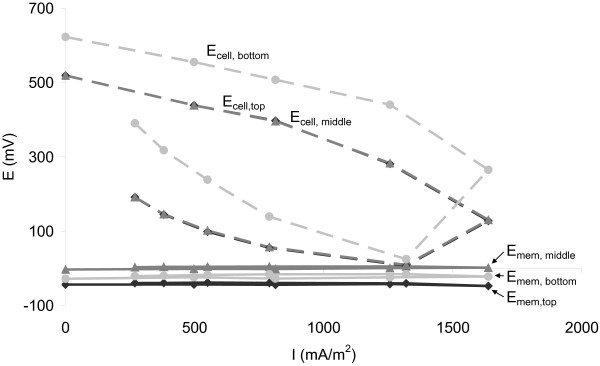
**Membrane voltages (E**_**mem**_**) and cell voltages (E**_**cell**_**) as a function of current density in top, middle and bottom sections of P-MFC 2 during polarization curve on day 320, in which membrane voltages stay low with increasing current, indicating a small membrane resistance.**

The high anode resistance in our experiment is in contrast with earlier research with the tubular system, which showed a high membrane resistance, which was mainly caused by the transport resistance [11,14]. In our experiment, however, membrane resistance stayed low during polarization (Figure 5), thus transport resistance did not increase. Experimental conditions of Timmers et al. differed on three important aspects from our experiment: 1) design, 2) plant-growth medium and 3) time-steps in the polarization curve.

### Influence of design on internal resistance

It is likely that the design of the system (tubular versus flat-plate) has a large influence on the internal resistance. In our experiment plant-roots were mainly found in the top part of the system, which was consistent with previous experiments [9,11,12,14]. This means that in the tubular system the average distance for protons produced in the anode (or other cations like K^+^ or Na^+^) to travel to the cathode is larger than in the flat-plate system (Figure 2). A larger distance will lead to a higher ionic resistance (Equation 8) and with that to a higher membrane resistance. It was shown in previous research by Timmers et al. [11,14] that membrane resistance was high and increased during polarization curves, which was not the case with the flat-plate system (Figure 5).

Internal resistance in the flat-plate P-MFC was, however, not lower than in the tubular P-MFC (Table 1). Current density was not higher in the flat-plate system P-MFC compared to the tubular P-MFC when normalized to membrane surface area (Table 1). The flat-plate design has, however, advantages over the tubular design. When calculating current and power per m^3^ it shows that Timmers et al. produced 7.5 A/m^3^ and 1.3 W/m^3^ whereas we produced 122 A/m^3^ and 5.8 W/m^3^. Firstly, this leads to the conclusion that materials are more efficiently used in the flat-plate system than in the tubular system, leading to higher outputs on volume base. Secondly, when normalizing current and power density to geographic planting area 1.6 A/m^2^ and 0.44 W/m^2^ were achieved, which is higher than reported for a tubular P-MFC (0.15 A/m^2^ and 0.22 W/m^2^[9,11]) in a polarization curve with 10 minute time-steps. So with less materials the flat-plate P-MFC harvests more electrons per plant than the tubular P-MFC and is therefore an important step towards optimizing the P-MFC.

**Table 1:** **Total internal resistances in Ω.m**^**2 **^**membrane and Ω.m**^**3 **^**reactor volume of P-MFC 1 and 2 (flat-plate) and P-MFC (tubular) of Timmers et al. 2012 [**[14]**]**

	**Time-step polarization curve (min)**	**Current density (mA/m**^**2**^**)**	**Total internal resistance (Ωm**^**2**^**)**	**Total internal resistance (Ωm**^**3**^**)**
P-MFC 1 top	10	58	1.4	0.03
P-MFC 1 top	10	169	2.4	0.05
P-MFC 2 top	10	54	2.4	0.05
P-MFC 2 top	10	143	3.1	0.06
P-MFC 1 middle	10	44	2.4	0.05
P-MFC 1 middle	10	91	4.2	0.08
P-MFC 2 middle	10	54	2.3	0.05
P-MFC 2 middle	10	143	3.0	0.06
P-MFC 1 bottom	10	29	3.5	0.07
P-MFC 1 bottom	10	33	9.7	0.19
P-MFC 2 bottom	10	68	1.4	0.03
P-MFC 2 bottom	10	295	1.3	0.03
Timmers et al. tubular	5	70	0.8	0.24
Timmers et al. tubular	5	1800	0.2	0.06
Timmers et al. Tubular	60	200	0.3	0.09
Timmers et al. Tubular	60	900	0.6	0.17

Numbers of Timmers et al. were estimated from figures.

### Influence of difference in plant-growth medium on internal resistance

In the tubular system a large transport resistance was found, whereas transport resistance in the flat-plate system is low. It should be noted though, that the plant-growth medium can influence transport resistance as well. Conductivity was 0.15-0.17 S.m^-1^ in the experiment of Timmers et al. and 1.1-1.3 S.m^-1^ in our experiment. Higher conductivity in our experiment will lead to a lower ionic resistance compared to Timmers et al. This would mean, however, that according to Equation 5 transport resistance could be expected to be higher than in the case of Timmers et al. The use of buffer in the experiment of Timmers et al. and the lack of it in our experiment can have an influence on the pH-gradient over the membrane. It is shown in Figure 4 that pH-resistance in our case can be positive or negative, meaning the pH-gradient from cathode to anode can be either positive or negative (Equation 6). When cathode-pH is higher than anode-pH, pH-gradient is positive, protons produced in the anode will have to migrate from anode to cathode. Protons migrate from a low pH to a high pH, so transport is driven by pH-difference. A pH-resistance is present, however, due to the change in electromotive force (EMF), as described by the Nernst equation (Equation 7). When the gradient is negative, however, protons will migrate from cathode to anode and the resistance is reversed to a driving force to produce protons. Based on Equation 5, a positive pH-resistance will reduce the transport resistance, but a negative pH-resistance will increase the transport resistance, which happens in the middle and bottom level of P-MFC 1 (Figure 5). Compared to the experiment of Timmers et al. transport resistance is lower in our experiment in all cases except bottom part of P-MFC 1. Thus, even though the difference in plant-growth medium between the experiments of Timmers et al. and ours could influence the internal resistance, it doesn’t seem to explain the reduction of transport resistance in the flat-plate system compared to the tubular system.

### Influence of time-steps used in polarization curve on internal resistance

Timmers et al. have shown that the time-steps used to make a polarization curve have a large influence on the internal resistance [14]. The longer the time-steps, the higher the internal resistance, due to an increasing anode resistance caused by proton build-up and an increasing membrane resistance caused by accumulation of cations in the anode [14]. In order to properly compare our results with those of Timmers et al. and understand the mechanisms, it is therefore important to realize that time-steps in the experiment of Timmers et al. (2011) were 5 or 60 minutes [14]. In Timmers et al. (2010) time-steps of polarization are not reported [11]. In our experiment time-steps of 10 minutes were used. Compared to the 5 minute time-step results of Timmers et al., a higher internal resistance could be expected in the flat-plate system, but compared to the 60 minute time-step results, our internal resistance was expected to be lower. Total internal resistance in the flat-plate P-MFC was higher than in the tubular system when normalized to membrane surface area, which cannot be explained from the difference in time-steps (Table 1). When we look to internal resistance when normalized to MFC-volume, however, the picture is different and the flat-plate P-MFC does indeed show a lower internal resistance than the tubular P-MFC in all cases except bottom anode of P-MFC 2 at low external resistance.

### Internal resistance in relation to power output of the flat-plate P-MFC

When comparing the obtained power and current densities of the flat-plate P-MFCs with the expected results based on Equation 1, it shows that only in a few cases measured value approximates theoretical value (Table 2). This has several reasons. Figure 1 was drawn based on theoretical potentials of anode and cathode and a total theoretical cell voltage of 1.1 V [20]. This can only be obtained, however, when using oxygen as final electron acceptor in the cathode, whereas we used ferric-cyanide, which has a lower theoretical potential than oxygen. Even so, use of oxygen would not directly lead to higher power outputs, since oxygen-use in the cathode usually leads to high cathode over-potentials due to limited diffusion of oxygen into the electrode and thus oxygen-limitation at the electrode surface. On the anode-side the theoretical potential is probably being overestimated when assuming that it is −0.5 V (vs Ag/AgCl), which is the theoretical potential of acetate oxidation under MFC-conditions [17]. Since substrate in the P-MFC originates from the plant, it is a complex mixture of several different organic compounds with different theoretical potentials [10]. Moreover, oxygen is being transported into the anode by the plant-roots, limiting the number of electrons available for electricity production, and, at low substrate concentrations leading to a mixed anode potential. This mixed anode potential will be higher than the assumed −0.5 V. Furthermore, the polarization curve shows that the internal resistance of the system is not linear. The theoretical internal resistance and resulting current and power output based on Equation 1 is only valid when internal resistance of the system is linear [21]. It is therefore not surprising that measured current and power densities don’t match with the theoretical values.

**Table 2:** **Expected and measured power densities (P, W/m**^**2**^**), based on calculated internal resistances (R**_**i**_**, Ωm**^**2**^**) and measured current densities (i, A/m**^**2**^**)**

**R**_**i **_**(Ωm**^**2**^**)**	**i (A/m**^**2**^**)**	**Expected P (W/m**^**2**^**)**	**Measured P (W/m**^**2**^**)**	**Δ P (W/m**^**2**^**)**
1.283	0.295	0.213	0.244	0.032
1.380	0.069	0.069	0.013	−0.056
1.444	0.058	0.059	0.010	−0.050
2.302	0.054	0.053	0.008	−0.045
2.354	0.054	0.053	0.008	−0.044
2.396	0.169	0.118	0.081	−0.037
2.404	0.044	0.043	0.005	−0.038
2.982	0.145	0.097	0.059	−0.038
3.060	0.142	0.095	0.057	−0.038
3.542	0.029	0.029	0.002	−0.027
4.169	0.091	0.066	0.023	−0.042
9.668	0.033	0.026	0.003	−0.023

## Conclusions

The flat-plate P-MFC design resulted in a lower transport and membrane resistance than the previously used tubular P-MFC. It did, however, not result in a lower total internal resistance normalized to membrane surface area. Total internal resistance of the flat-plate P-MFC is at best comparable to total internal resistance of the tubular P-MFC, only differently distributed over several partial internal resistances. In the flat-plate P-MFC the anodic resistance is highest, when using a chemical cathode, due to substrate limitation or mass transfer limitation. To overcome the problem of substrate limitation, either the plant should exudate more, exudates should be converted into electricity more efficiently or other rhizodeposits, like dead root material should be used. Possibly, when rhizosphere is fully mature, more dead root material is available, which will probably lead to higher substrate availability in the P-MFC. Higher exudation rates might be achieved by adapting the plant-growth medium to stimulate exudation [22]. For future research it would be interesting to further reduce anode height, since the middle and bottom level anodes generally generated less electricity (Figure 3) than the top one and most roots were found in the top anode.

## Materials and methods

### Set-up and operation

**Figure 6:**
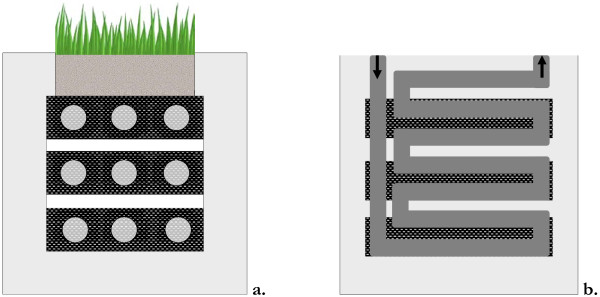
**Anode (6a) and cathode (6b) schematic overview of a flat-plate Plant Microbial Fuel Cell.** In anode and cathode top, middle and bottom sections are shown; in anode sample-points are shown; and in cathode the flow-through channel is shown

Two flat-plate P-MFCs were run for 703 days. The anode compartment had a total volume of 648 ml (18x18x2 cm). The anode consisted of three sections of graphite felt of 5 cm height (Grade WDF, 6 mm, National Specialty Products Carbon and Graphite Felt, Taiwan), which were physically separated (Figure 6a) A gold wire was woven through the graphite felt of each section to serve as current collector. Three plants of *Spartina anglica* (grown in a greenhouse from offshoot of Timmers et al. (2010)) were planted in each P-MFC. The anode compartment was separated from the cathode compartment by a cation exchange membrane (CEM) (Fumatec, Frankfurt, Germany).

The cathode consisted of three sections of graphite felt of 5 cm height, (Grade WDF, 2.8 mm, National Specialty Products Carbon and Graphite Felt, Taiwan), each corresponding with an anode section (Figure 6b). The cathode compartment consisted of two flow-through modules with the graphite felt in between. A gold wire was woven through the graphite felt of each section to serve as current collector. From day 70 ferric cyanide (K_3_Fe(CN)_6_, 50 mM) was used as catholyte to stabilize cathode-potential and be able to study anode-potential. Ferric cyanide was replenished whenever cathode-potential dropped below +200 mV (against Ag/AgCl reference electrode).

From day 1 through day 116 top, middle and bottom sections of the anode were electronically connected, thus behaving as one anode. During this period external load between anode and cathode was 300 Ω. From day 117 anode-sections were electronically disconnected. The three anode-sections all were connected to their corresponding cathode over an external load of 900 Ω.

Different plant-growth media were used during the runtime of the experiment, as described in Helder et al. 2011 for P-MFC 3 and 4 [13]. At determination of the internal resistances at day 320 a nitrate-less, ammonium-rich medium was used with composition as in Table 3.

**Table 3:** Concentrations of macronutrients, micronutrients, and added salts in demineralized water as composition of the plant-growth medium

	**Concentration**
**Macronutrients**	**mg/L**
NH_4_HCO_3_	553.5
CaCl_2_	222
NH_4_H_2_PO_4_	115.1
MgSO_4_.7H_2_O	123.245
Na_2_SiO_3_.9H_2_O	142.1
KCl	223.68
**Micronutrients**	**mg/L**
KCl	1.864
H_3_BO_3_	0.773
MnSO_4_.H_2_O	0.169
ZnSO_4_.7H_2_O	0.288
CuSO_4_.5H_2_O	0.062
H_2_MoO_4_ (85 % MoO_3_)	0.04
CoCl_2_.6H_2_O	1
Na_2_SeO_3_	0.05
C_14_H_18_N_3_O_10_FeHNa	5
NiSO_4_.6H_2_O	0.03
**Salt**	**g/L**
NaCl	10

Anode and cathode potential were measured with Ag/AgCl-reference electrodes (3 M KCl, + 205 mV versus SHE, ProSenseQis). Data was logged every 60 seconds with Fieldpoint (Module S, National Instruments) and collected with Labview (National Instruments Software). To test maximum power density polarization curves were made simultaneously for each section. These were made by disconnecting anode and cathode for 30 minutes to reach open cell voltage (OCV) and subsequently connecting anode and cathode over an external load of 900, 500, 250, 100, 10, 100, 250, 500 and 900 Ω, for 10 minutes each. At day 320 anode was sampled at 9 sample-points, which were evenly distributed over the three anode-sections, so three sample-points in each anode-section. Samples of 1 ml each were measured for conductivity (ProLine Plus Qis, ProSense BV, Oosterhout, The Netherlands) and pH (691 pH-meter, Metrohm, Herisau, Switzerland).

The set-ups were placed in a climate chamber (Microclima 1750 Snijders) with an average light intensity of 596 ± 161 μmole m^-2^ s^-1^, measured at the top of the set-ups witha light intensity meter (Photodyne 44XLA). The climate chamber was controlled at 25°C with 75% humidityand a day-night regime of 14:10 hours was used.

After 703 days the set-ups were taken apart and root-weight was measured for the three anode-sections per P-MFC.

### Calculations

Internal resistance of the P-MFC can be split in several partial internal resistances as in [23]:

(2) $$R_{\mathrm{int}}=R_{\mathrm{an}}+R_{\mathrm{cat}}+R_{T}$$

Equation 2 Internal resistance in a P-MFC (Ω.m^2^), in which *R*_*an*_ = anodic resistance (Ω.m^2^), *R*_*cat*_ = cathodic resistance (Ω.m^2^), *R*_*T*_ = transport resistance (Ω.m^2^).

Anodic resistance can be calculated from the over-potential of the anode according to [14]:

(3) $$R_{\mathrm{an}}=\frac{\left(E_{\mathrm{an}}-E_{\mathrm{an}}^{0}\right)}{i}$$

Equation 3 Anodic resistance in a P-MFC (Ω.m^2^), in which $E_{\mathrm{an}}^{0}$ = theoretical anode potential (V), *E*_*an*_ = measured anode potential at a certain external resistance (V), *i* = current density (A/m^2^).

Since the theoretical anode potential in the P-MFC is based on a mixed potential [21] and concentrations of different exudates are unknown, it is assumed that theoretical anode potential equals open cell potential (E_an,OCP_).

Cathodic resistance can be calculated from the cathode over-potential according to [14]:

(4) $$R_{\mathrm{cat}}=\frac{\left(E_{\mathrm{cat}}^{0}-E_{\mathrm{cat}}\right)}{i}$$

Equation 4 Cathodic resistance in a P-MFC (Ω.m^2^), in which $E_{\mathrm{an}}^{0}$ = theoretical cathode potential at 50 mM ferric cyanide solution (V), *E*_*cat*_ = measured cathode potential at a certain external resistance (V), *i* = current density (A/m^2^).

Transport losses of the system were calculated as in Timmers et al. (2010) [11] and can be calculated as [11,23]:

(5) $$E_{T}=E_{\mathrm{mem}}-E_{\mathrm{\Delta pH}}-E_{\mathrm{ionic}}$$

Equation 5 Transport loss in a P-MFC (V), in which; E_mem_ = measured potential over the membrane (V), E_ΔpH_ = pH gradient loss (V), E_ionic_ = ionic loss (V).

When dividing this equation by current, transport resistance can be calculated. The potential over the membrane is measured as the difference between the reference electrode in the anode and the reference electrode in the cathode. The pH resistance is calculated as [11]:

(6) $$R_{\mathrm{\Delta pH}}=\frac{\left(\frac{RT}{F}\ln\left(10^{\left(pH_{\mathrm{cath}}-pH_{\mathrm{an}}\right)}\right)\right)}{i}$$

Equation 6 pH resistance in an MFC, in which pH_cath_ = cathode pH, pH_an_ = anode pH, and i = current density per membrane area.

Equation for pH resistance is derived from the Nernst equation:

(7) $$E_{OCP,an}=E_{\mathrm{an}}^{0}-\frac{RT}{nF}\ln\frac{\left[CH_{3}COO^{-}\right]}{{\left[H^{+}\right]}^{9}{\left[HCO_{3}^{-}\right]}^{2}}$$

Equation 7 Nernst-equation, in which $E_{\mathrm{an}}^{0}$ is the standard potential (V), R is the universal gas constant (8.314 J mol-1 K-1), T is the temperature (K), n is the number of electrons involved in the reaction (−), F is Faraday’s constant (96485 C mol-1), $\left[CH_{3}COO^{-}\right]$ is the acetate activity (mol L-1), $\left[H^{+}\right]$ is the proton activity (mol L-1), and $\left[HCO_{3}^{-}\right]$ is the bicarbonate activity (mol L-1).

This equation shows that one pH-unit difference will lead to a change in anode potential of 59 mV.

Ionic resistance is calculated as [11,23]:

(8) $$R_{\mathrm{ionic}}=\left(\frac{d_{\mathrm{an}}}{\sigma_{\mathrm{an}}}\right)$$

Equation 8 Ionic resistance in the anode of an MFC, in which d_an_ = average distance between point of proton production and membrane, and σ_an_ = conductivity of anolyte.

Power densities are expressed per geometric planting area because it gives insight in the possibility to use this technology for large scale electricity production.

## Abbreviations

P-MFC: Plant-Microbial Fuel Cell the technology to produce electricity with living plants; MFC: Microbial Fuel Cell bio-electrochemical system to produce electricity from organic matter; EMF: Electromotive force driving force for electrons; CEM: Cation exchange membrane selective membrane for cations; OCV: Open Cell Voltage voltage at open circuit (zero current).

## Competing interests

Two of the authors of the manuscript, Marjolein Helder and David Strik, founded a spin-off company from Wageningen University to further develop the Plant-Microbial Fuel Cell into products. The company does not generate any income and has no interest in the conducted research. Results presented in this paper were not in any way influenced by the existence of the spin-off company.

## Authors’ contributions

MH co-designed and co-executed the experiment, analyzed data and drafted the paper, DS co-designed and co-executed the experiment, critically assessed data and critically reviewed the paper, HH invented the technology, co-designed the experiment, critically assessed data and critically reviewed the paper, CB designed the research project, critically assessed data and critically reviewed the paper. All authors read and approved the final manuscript.
